# Serum‐Derived Extracellular Vesicles as Biological Indicator of Mobility Resilience in Older Adults

**DOI:** 10.1111/acel.70470

**Published:** 2026-04-06

**Authors:** Nicholas F. Fitz, Ashish Kumar, Yixin Su, Mitu Sharma, Sangeeta Singh, Radosveta Koldamova, Iliya Lefterov, Amrita Sahu, Fabrisia Ambrosio, Caterina Rosano, Gagan Deep

**Affiliations:** ^1^ Department of Environmental & Occupational Health, School of Public Health University of Pittsburgh Pittsburgh Pennsylvania USA; ^2^ Department of Internal Medicine‐Gerontology and Geriatric Medicine Wake Forest University School of Medicine Winston‐Salem North Carolina USA; ^3^ Department of Physical Medicine and Rehabilitation University of Pittsburgh Pittsburgh Pennsylvania USA; ^4^ McGowan Institute of Regenerative Medicine University of Pittsburgh Pittsburgh Pennsylvania USA; ^5^ Discovery Center for Musculoskeletal Recovery Schoen Adams Research Institute at Spaulding Charlestown Massachusetts USA; ^6^ Department of Physical Medicine & Rehabilitation Harvard Medical School Boston Massachusetts USA; ^7^ Department of Physical Medicine & Rehabilitation Spaulding Rehab Hospital Charlestown Massachusetts USA; ^8^ Department of Epidemiology, School of Public Health University of Pittsburgh Pittsburgh Pennsylvania USA; ^9^ Atrium Health Wake Forest Baptist Comprehensive Cancer Center Wake Forest University School of Medicine Winston‐Salem North Carolina USA; ^10^ Sticht Center for Healthy Aging and Alzheimer's Prevention Wake Forest University School of Medicine Winston‐Salem North Carolina USA

**Keywords:** extracellular vesicles, mitochondria, mobility resilience, skeletal muscle, small noncoding RNAs

## Abstract

Mobility decline with aging is a major health concern, associated with a higher risk for disability. Despite the prevalence of gait slowing in elderly adults, this issue has not been adequately addressed. The central nervous and skeletal muscle systems are key regulators of gait speed. However, direct molecular communication along the brain‐muscle axis and their interactions in mobility resilience remain poorly studied. Extracellular vesicles (EVs) have emerged as a key player in long‐distance inter‐cellular communication. Nevertheless, the potential of EVs as biological predictors of mobility resilience in older adults has not been studied. We used serum from 23 participants with gait speed > 1.0 m/s (resilient) and 22 participants with gait < 1.0 m/s (non‐resilient) from the Health, Aging and Body Composition (Health ABC) study. Total circulating serum EVs were isolated and small noncoding RNAs characterized using un‐biased sequencing. Given the central role of mitochondria in muscle energy metabolism and link to age‐related physical decline, next, muscle‐derived EVs (MDE) were isolated and characterized for mitochondrial markers (TOM20, mtCox2, PDH, and VDAC) by flow cytometry, 13 miRNAs related to mitochondrial function by RT‐PCR, and PPAR‐γ by ELISA. The results showed differential enrichment of various miRNAs, circRNAs, and mitochondrial proteins in total EVs and/or MDE between mobility resilient and non‐resilient groups, highlighting their potential as non‐invasive biomarkers for mobility outcomes. Overall, these findings suggest a role for serum EVs in mediating molecular communication related to functional aging phenotypes and underscore the potential of EV biomarkers in modulating mobility and promoting healthy aging.

## Graphical abstract

We show unique signatures of small noncoding RNA cargos and mitochondrial proteins in serum EVs and muscle‐derived EVs associated with age‐related mobility resilience. These findings suggest a role for serum EVs in mediating molecular communication related to functional aging phenotypes and underscore the potential of EV biomarkers.

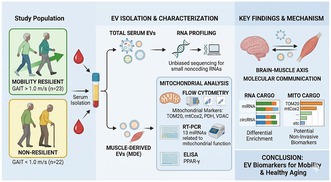

**Abbreviations**
3MS: modified mini‐mental status exam
AD: Alzheimer's disease
BBB: blood–brain barrier
circRNAs: circular RNAs
CNS: central nervous system
DSST: digit symbol substitution test
EVs: extracellular vesicles
MDE: muscle‐derived EVs
miRNAs: microRNAs
mtCox2: cytochrome c oxidase subunit 2
ncRNA: non‐coding RNA
NTA: nanoparticle tracking analysis
PDH: pyruvate dehydrogenase
piRNAs: PIWI‐interacting RNAs
PPAR‐γ: peroxisome proliferator‐activated receptor gamma
SGCA: α‐sarcoglycan
SMS: skeletal muscle system
snoRNA: small nucleolar RNAs
snRNAs: small nuclear RNAs
TEM: transmission electron microscopy
TOM20: translocase of outer mitochondrial membrane 20
tRNAs: transfer RNAs
VDAC: voltage‐dependent anion channel

## 1 Introduction

Slow walking gait with aging is a major health concern linked to increased risk of frailty and dementia, loss of independence, increased health care use, and reduced survival (Studenski et al. 2011). With aging, elderly adults (> 70 years) can develop slow gait in the absence of a clear diagnosis or cause. We showed that ~80% of older adults with more than one locomotor risk factor (e.g., muscle weakness, joint pain) have mobility decline and considered mobility non‐resilient (gait speed < 1.0 m/s), which is associated with high risk for disability (Rosso et al. 2017). Although other individuals with similar locomotor risk factors maintain mobility resilient (gait speed > 1.0 m/s) with aging. Despite the high prevalence of gait slowing in elderly, this decline in mobility and activity is inadequately addressed and underlying mechanisms are poorly understood. Although slow walking is widely recognized as a multisystemic phenomenon, alterations in the central nervous system (CNS) and skeletal muscle system (SMS) have emerged as significant predictors of gait speed and point to a brain‐muscle axis that plays a critical role in mobility resilient. These systems exert critical modulatory and interactive influences on mobility performance, independent of the severity of underlying locomotor risk factors. The coordination, integrity, and functional capacity of the CNS and SMS play a pivotal role in maintaining faster gait and overall mobility, even amidst age‐related declines in other systems. Notably, both the CNS and SMS remain adaptable later in life and influence each other through neural pathways and circulating biological factors, making them promising targets for interventions aimed at sustaining mobility with age.

Although there are direct nerve connections between the brain and skeletal muscles via motor and sensory axons, other pathways are also important in the brain‐muscle axis. One potential candidate to coordinate brain and muscle communication is extracellular vesicles (EVs). EVs are lipid membrane‐delimited nanoscale vesicles secreted by almost all cells into the interstitial space and contain specific cargos including nucleic acids, proteins, metabolites, and lipids (Fitz et al. 2021; Kumar et al. 2022, 2023; Mishra et al. 2025; Sahu et al. 2021). EVs are heterogeneous in size and can be categorized as small EVs (~50–200 nm), large EVs (200–1000 nm), and very large EVs (1–10 μm) (Welsh et al. 2024). Due to the nano size of small EVs, they can travel long‐distance via circulatory system and could play a critical role in exchanging molecular information between remote sites in the body. EVs cargoes, taken up by target cells, can regulate physiological functions or pathological processes in recipient cells. Importantly, the content of EVs, at the core and/or the surface allows identification of organ specificity including brain or muscle specific EVs (Cleary, Kumar, Craft, et al. 2025; Kumar et al. 2024). In multiple studies, we have reported the utility of EVs for examining the pathophysiological state of different cells and tissues including brain cells and muscle tissues (Cleary, Kumar, Craft, et al. 2025; Kumar et al. 2021, 2024, 2022, 2023; Mishra et al. 2025). Notably EVs, regardless of their origin, are enriched with extracellular RNAs of many different types, particularly small noncoding RNAs (ncRNAs). The most frequently reported types of small ncRNAs carried by small EVs include: miRNAs (MicroRNAs), tRNAs (Transfer RNAs), piRNAs (PIWI‐interacting RNAs), snRNAs (Small nuclear RNAs), snoRNA (Small nucleolar RNAs), and circRNAs (Circular RNAs). Although the exact functions of various small ncRNAs are not fully known, studies have shown their ability to regulate gene expression at the level of posttranscriptional messenger RNA (mRNA) processing (Becker et al. 2019; Gebert and MacRae 2019; Telonis et al. 2015). For example, miRNAs bind to a specific sequence of target mRNAs to induce translation repression through either mRNA silencing or degradation. In contrast, circRNAs can act as microRNA sponges, inhibiting miRNA modulation of gene expression (Li et al. 2021). Therefore, circulating EVs carry small ncRNAs which can act either as activators or suppressors of gene expression in a complex relationship (Barros et al. 2018).

It has been proposed that physical exercise and the skeletal muscle secretome reduces age‐related brain atrophy, oxidative stress, neuroinflammation, and neurodegenerative disease progression while supporting cognitive function and mitochondrial biogenesis (Cotman and Berchtold 2007; Delezie and Handschin 2018; Fitz et al. 2023; Lin et al. 2026; Marosi et al. 2012; Sahu et al. 2021; Steiner et al. 2011). Circulating factors such as myokines, growth factors, hormones, and cytokines have been proposed as mediators of this brain‐muscle communication (Delezie and Handschin 2018; Kostka et al. 2024). However, their ability to influence central processes may be constrained by limited permeability across the blood–brain barrier (BBB). We and others showed that EVs are key messengers of paracrine exercise signals (Murphy et al. 2020; Nederveen et al. 2020; Vechetti et al. 2021; Whitham et al. 2018). EVs possess the unique capability to traverse the BBB, enabling bidirectional signaling between brain and peripheral tissues through the delivery of bioactive cargo including small ncRNAs (Alvarez‐Erviti et al. 2011; Katsuda et al. 2013; Li et al. 2018; Morad et al. 2019). EVs may carry both neuroprotective and myoprotective factors capable of crossing the BBB, thereby linking central and peripheral healthy aging (Fitz et al. 2023; Kumar et al. 2024; Sahu et al. 2021). Also, muscle‐derived EVs (MDE) have been shown to have significant role in mediating the crosstalk between skeletal muscles and other tissues (Aswad et al. 2014; Jia et al. 2024). We have shown the utility of neuromuscular electrical stimulation (NMES) to promote the release of circulating EVs that have both myo‐ and neuro‐protective effects, which increased the ability to repair injured skeletal muscle in aged mice (Bean et al. 2023; Fitz et al. 2023). We also observed higher levels of neuroprotective molecules in EVs, for example, such as BDNF (Brain‐derived neurotrophic factor), following muscle contractile activity supporting that circulating EVs may serve as key mediators in the brain‐muscle axis (Bean et al. 2023).

We hypothesized that brain and skeletal muscle crosstalk not only regulates the functions of each system but ultimately influences gait speed. We further hypothesized that circulating EV cargos contain molecular messengers that are important in modulating mechanisms underlying the brain‐muscle axis. Although there is abundant data that the CNS and SMS have beneficial effects on each other, and that enhancing one can lead to improvements in the other and mobility, this evidence is primarily from preclinical models and studies of each system in isolation. For the purpose of this study, we wanted to determine the utility of total serum and muscle‐derived EVs and their relevant cargos as biological indicators of mobility resilience in older adults. Analysis of the circulatory EVs for their cargos, particularly small ncRNA, could be valuable due to their regulatory function in mediating gene expression and cellular signaling and can provide insight into the possible molecular signals and mechanisms facilitating communication between these two distant organs. Moreover, since mitochondria play a central role in muscle health, particularly through their functions in energy production, cellular maintenance, and adaptation to stress, mitochondrial proteins were studied in MDE to better understand the molecular mechanisms underlying age‐related declines in physical performance. We first conducted a comprehensive profiling of circulating total EVs for small ncRNA, followed by MDE cargos for mitochondrial markers using serum of age‐matched older individuals, classified as mobility resilient or non‐resilient. We identified unique small ncRNAs in EVs isolated from the serum of mobility resilient compared with non‐resilient individuals. Furthermore, many of the small ncRNAs have been shown to be important in regulating genes involved in brain health and metabolism. Further, the mitochondrial markers showed higher levels in MDE from the mobility resilient group. Utilizing linear regression modeling, we also show associations of these mitochondrial markers with mobility phenotypes in the cohort. These results can help inform future studies to determine the importance of EV cargos in pathological aging and potentially identify biomarkers which would allow for detection of early changes in mobility or monitor interventions to help improve mobility among elderly individuals.

## 2 Materials and Methods

### 2.1 Study Population

The Health, Aging and Body Composition (Health ABC) Study recruited participants (70–79 years) from Memphis, Tennessee, or Pittsburgh, Pennsylvania (Kanaya et al. 2009). Participants free of difficulty walking ¼ mile and climbing 10 stairs were eligible and followed from 1997 to 2011. We defined mobility resilient as at least one locomotor risk factor (pain, high BMI, low forced expiratory volume, peripheral artery disease) and a gait speed > 1.0 m/s, whereas non‐resilient individuals were defined as one locomotor risk factor and gait speed < 1.0 m/s (Rosso et al. 2017). We randomly selected 23 resilient participants and 22 non‐resilient. Blood samples (11 resilient, 10 non‐resilient) were assessed at Wake Forest School of Medicine (WFSM, Site one) and (*n* = 12 each) University of Pittsburgh (UPitt, Site two). Blood samples were collected at the year two visit in the morning after overnight fasting for at least 8 h and serum stored at −80°C. All participants were provided with written informed consent, and protocols were approved by the institutional review board at the University of Tennessee, Memphis, TN, and University of Pittsburgh, Pittsburgh, PA.

Measures of interest included age, sex, race, cognitive function, muscle strength and comorbidities measured as previously described (Health ABC 2025) (https://healthabc.nia.nih.gov/). Presence of comorbidities was assessed via self‐reported questionnaires, medications listed, or Health Care Finance Administration diagnosis. Digit symbol substitution test (DSST, range 0–100) tested executive function and information processing skills, with higher scores indicating better executive functioning (Barha et al. 2019). Modified mini‐mental exam (3MS, range 0–90) is widely used as a marker of cognitive change and incident dementia in older adults (Kuller et al. 2003); with higher scores indicating better function (Andrews et al. 2019). Muscle strength was measured as right knee extension strength on an isokinetic dynamometer (Kin‐Com dynamometer); the left leg was tested for those with right joint replacement or knee pain. Other contraindications to strength testing included: systolic blood pressure ≥ 200 mmHg, diastolic blood pressure ≥ 110 mmHg, history of cerebral aneurysm, cerebral bleeding, bilateral total knee replacement, or severe bilateral knee pain (12.7% of cohort). Maximum muscle torque was calculated from the average of three reproducible and acceptable trials from a maximum of six.

### 2.2 Isolation of Total Serum EV

Serum EVs were isolated using Plasma/Serum Exosome Purification Mini Kit (Norgen Biotech). Four hundred microliter serum was used in which 3.6 mL particle‐free water was added. To each sample, 100 μL of ExoC buffer was added followed by 200 μL of Slurry E and incubated at room temperature for 5 min. Samples were then centrifuged for 2 min at 2000 RPM; the supernatant was removed and 200 μL ExoR buffer added. After incubation for 5 min, samples were centrifuged for 2 min at 500 RPM and supernatant transferred to filter spin column and centrifuged for 1 min at 6000 RPM to isolate EVs.

### 2.3 Isolation of Muscle‐Derived EVs (MDE)

Isolation of MDE was performed on total serum EVs using α‐sarcoglycan (SGCA) marker as before (Mishra et al. 2025). Briefly, 1500 μg of total serum EVs were incubated with 8 μg of biotin‐tagged anti‐SGCA antibody (orb453551; Biorbyt) overnight at 4°C with continuous mixing. Streptavidin‐tagged magnetic beads were then added for 2 h at room temperature with mixing. Beads were washed 3 times with 0.1% Tween‐20 in Tris‐buffered saline and MDE was eluted with 200 μL of IgG elution buffer (21004; Thermo Fisher). Beads were magnetically removed, and MDE supernatant was collected in tubes containing 20 μL of 1 M tris buffer (pH 9) to neutralize the pH.

### 2.4 EV Characterization

For EV characterization, 1 mL of diluted EV sample (1:500) was loaded on a NS300 Nanosight (Malvern Instruments) at a rate of 400 μL/min. Particle‐free PBS was used to wash the instrument between samples. Sample analysis consisted of 3 × 30 s videos. Nanoparticle Tracking Analysis (NTA) software (v. 3.4) was used to measure particle size and concentration. EV samples were further characterized by Western blotting and transmission electron microscopy (TEM). For Western blotting, EV proteins were resolved on 4%–12% Bis‐Tris gels (Thermo Fisher) and transferred onto nitrocellulose membranes (IB23001; Thermo Fisher, iBlot2). These membranes were probed with anti‐CD63 (System Biosciences EXOAB‐CD63A‐1, 1:500) and anti‐CD81 antibodies (EXOAB‐CD81A‐1, 1:500). Immunoreactive signals were visualized with chemiluminescence on an Amersham Imager 600 (GE Lifescience). TEM was performed on EVs as described (Fitz et al. 2021). Briefly, EVs were adhered to copper grids coated with 0.125% Formvar in chloroform, stained with 1% uranyl acetate in ddH_2_O, and imaged immediately using a JEM 1011.

### 2.5 Flow Cytometry

To analyze surface levels of mitochondrial markers, total EVs and MDE were incubated with fluorescently labeled translocase of outer mitochondrial membrane 20 (TOM20), voltage‐dependent anion channel (VDAC), cytochrome c oxidase subunit 2 (mtCox2), or pyruvate dehydrogenase (PDH) antibodies overnight at 4°C. Further, EVs were labeled with membrane labeling dye CellBrite 488 (Biotium) for 15 min. EVs without membrane labeling dye were used to set gates for dye positive events. Dye labeled EVs without antibodies were used to set gates for antibody positive events. Samples were analyzed on a nano‐flow cytometer (CytoFlex, Beckman Coulter) as described (Kumar et al. 2023; Mishra et al. 2025).

### 2.6 RNA Isolation and Library Preparation

**FIGURE 1:**
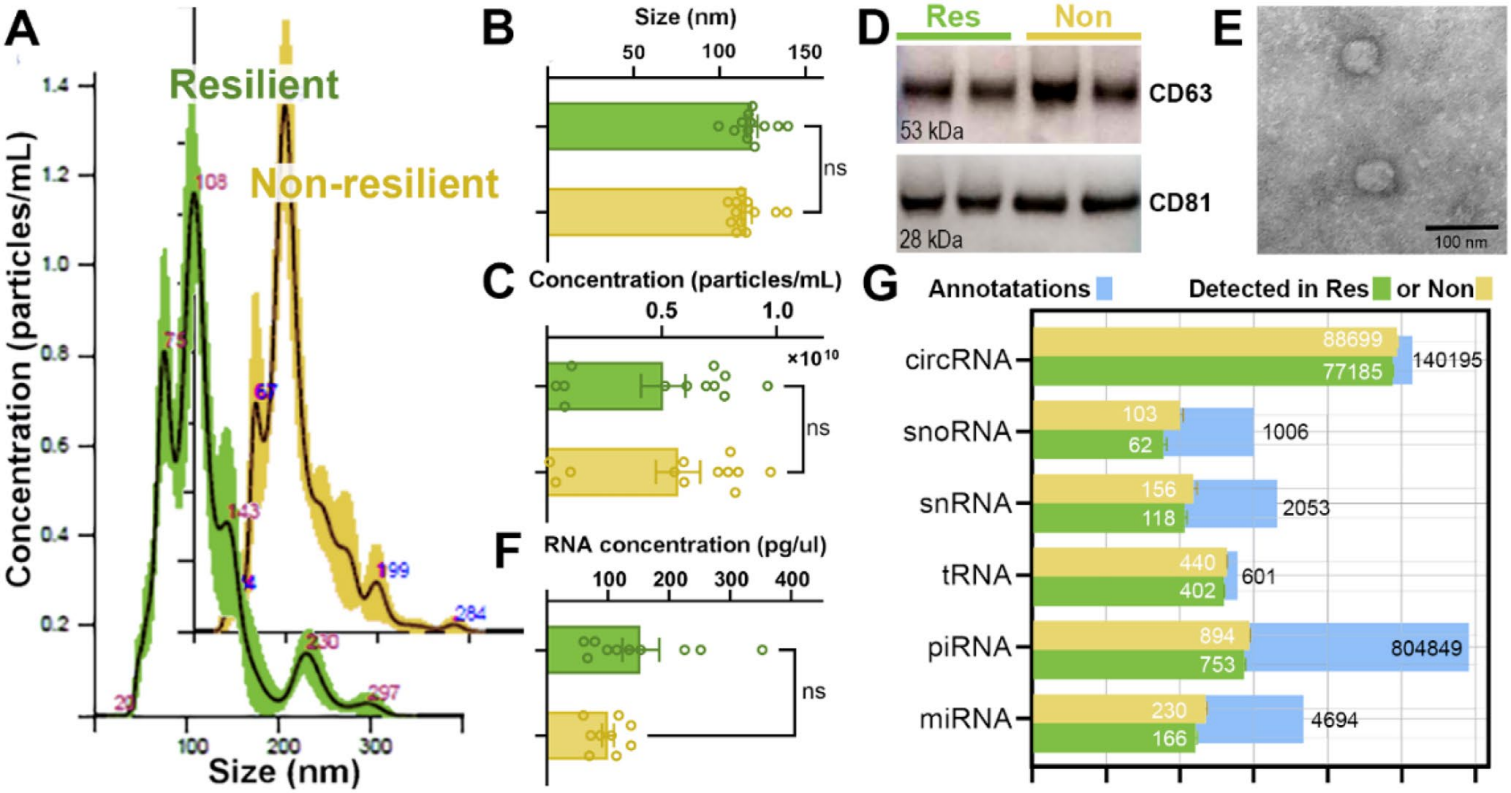
Characterization and quantification of total circulating EVs isolated from serum of mobility resilient and non‐resilient individuals. (A) Isolated total circulating serum EV particle size distribution profiles were measured by NTA from resilient (Res, green) and non‐resilient (Non, yellow) individuals. Bar graphs representing EV size (B) and concentration (C), as measured by NTA analysis for serum EV. (D) Representative western blot images of classical EV markers CD63 and CD81 showing no difference between groups. (E) Representative TEM image to visualize size and morphology of serum EVs. (F) Bar graph showing no significant difference in the concentration of RNA isolated from serum EVs. (G) Bar plot showing the number of small ncRNA transcripts annotated from serum EVs of the resilient and non‐resilient groups. Blue bars represent the total number of possible annotations in COMPSRA. Note a high percentage of annotated small ncRNAs and no significant difference between groups. Bar graphs represent mean ± SEM; Statistical significance was determined by unpaired *t*‐test. ns, not significant. *n* = 12.

Total RNA was extracted from serum EVs using Exosomal RNA Isolation Kit (Norgen Biotech). Quantification and quality of RNA were evaluated with Agilent 2100 Bioanalyzer RNA Pico assay (Agilent Technologies) and no significant difference in RNA amounts was observed between groups (Figure 1F). RNA was pelleted with Pellet Paint (Novagen) and reconstituted in 6 μL of nuclease free water, and the total amount of RNA isolated was used for small RNA library generation (average 125 ng/μL). The NEBNext small RNA sample library kit (New England Biolabs) was used with modifications of 1:10 adapter dilution and 18 PCR cycles. Libraries were cleaned using AMPure XP beads (Beckman Coulter), quality assessed with Bioanalyzer 2100 High Sensitivity DNA chip, and sequenced on the NextSeq 2000 (Illumina, 1 × 51 bp).

RNA isolation from MDE was performed using an equal volume of MDE, isolated from 1500 μg of total sEV, following the protocol reported by us earlier (Cleary, Kumar, Su, et al. 2025; Kumar et al. 2023; Mishra et al. 2025, 2023).

### 2.7 Computational Analysis of Sequencing Data

Small ncRNA library data generated from serum EVs and published data (Isakova et al. 2020) were aligned and quality checked by STAR (v 2.5.3a), annotated with COMPSRA (v 1.0.3) and differential abundance determined by DESeq2 (v 1.36.0). Selected microRNA gene targets were analyzed with TargetScan at cumulative weighted context score cutoff < −0.5. For circRNA, associated genes were identified by circBase (www.circbase.org). Functional annotation clustering was performed using Metascape (Zhou et al. 2019) with significance at *p* < 0.05.

### 2.8 MicroRNA Analysis in MDE

MDE were analyzed for the levels of 13 miRNAs (Let‐7e‐5p, miR‐100‐5p, miR‐101‐3p, miR‐125a‐5p, miR‐125b‐5p, miR‐27a‐3p, miR‐27b‐3p, miR‐29a‐3p, miR‐34a‐5p, miR‐378a‐3p, miR‐423‐5p, miR‐708‐5p) related to mitochondrial function, with real‐time PCR using TaqMan Advanced miRNA Assay (20×). Each RNA sample was mixed with 0.5 μL of cel‐miR‐39‐3p (Qiagen: Cat no. 339390) from a 0.0008 fmol/μL stock, which served as spike‐in normalization control. Isolation of total RNA and cDNA synthesis was performed with MDE as our published protocol (Kumar et al. 2023; Mishra et al. 2023). Prepared cDNA was diluted 3‐fold, and 1 μL used for qPCR analysis. An equal amount of total RNA was used for cDNA synthesis, and an equal volume of cDNA was used for qPCR analysis. Relative level of different miRNAs in each sample was normalized with cel‐miR‐39‐3p to calculate Δ*C*_t_ values. To analyze the MitoFunction score, the median value of each miRNA was determined and subtracted from the Δ*C*_t_ values (cel‐miR‐39 normalized) to obtain the median deviation. The change in Δ*C*_t_ value for each miRNA from the median (in any direction; increase or decrease) was scored as follows: 0–1 = score 0, 1–2 = score 1, 2–3 = score 2, more than 3 = score 3. The cumulative score from all miRNAs was presented as Mito‐Function score.

### 2.9 Enzyme‐Linked Immunosorbent Assay (ELISA)

Peroxisome proliferator‐activated receptor gamma (PPAR‐γ) levels in MDE were analyzed using ELISA (MyBiosource) after lysing with 10× RIPA. Protein levels of MDE lysate were measured by bicinchoninic acid assay (BCA) and the concentration of PPAR‐γ calculated from the standards after normalization with MDE protein concentration. PPAR‐γ levels in MDE were plotted in ng/mL/μg of MDE.

### 2.10 Statistical Analysis

Population characteristics were first compared between groups and then by resilience status, using independent *t*‐test and chi square when appropriate. EV characterization was analyzed with a two‐tailed unpaired *t*‐test (GraphPad Prism v10.0.3). Biological function terms were reduced and visualized utilizing REVIGO, reduce + visualize Gene Ontology (v 1.8.1). Comparisons of molecular cargo across groups were first unadjusted and then adjusted for demographics using linear regression models (SPSS 31.0).

## 3 Results

### 3.1 Health ABC Participant Characterization

Among the participants included in this analysis, 53% were women and 35% were Black, with an average age of ~74 years. Mobility resilient was defined as at least one locomotor risk factor (pain, high BMI, low forced expiratory volume, peripheral artery disease) and a gait speed > 1.0 m/s, whereas non‐resilient individuals were defined as one locomotor risk factor and gait speed < 1.0 m/s. Participants used for total serum circulating EVs and MDE did not significantly differ on any of the demographic characteristics (Table 1), consistent with the randomized assignment and supporting comparisons of study results. As expected, resilient status was related to faster gait speed (Table 2). Additionally, resilient was associated with higher performance in the two cognitive tests (DSST, 3MS), and muscle strength (Table 2); which has been related with gait speed in prior studies (Rosano et al. 2005, 2012). Additionally, there was a statistically significant association between resilient status and sex in the MDE study (resilient were more likely women, Table 2) and race in small ncRNA of total serum EVs (resilient were more likely white, Table 2), thus these variables were considered as covariates in the analyses of EV markers.

**TABLE 1:** Participants characteristics for the experiments conducted at Site 1—Wake Forest University and Site 2—University of Pittsburgh.

	Mean ± SD or *n* (%)		*p*
	Site 1 (*N* = 21)	Site 2 (*N* = 24)	
Age, years	73.05 ± 2.60	74.13 ± 3.43	0.24
Sex, women	11 (52)	13 (54)	0.91
Race, Non‐Hispanic White	14 (67)	14 (58)	0.76
Gait speed, m/s	1.30 ± 0.56	1.07 ± 0.27	0.11
Comorbidities, number	1.81 ± 0.75	2.05 ± 1.09	0.41
Time spent walking (min/week)	178.81 ± 276.64	94.62 ± 149.68	0.23
Digit symbol substitution test, points	34.38 ± 12.53	35.25 ± 19.84	0.86
Modified mini‐mental status exam, points	90.43 ± 5.91	86.92 ± 10.40	0.17
Muscle strength, Nm	92.34 ± 34.02	82.94 ± 34.73	0.42

**TABLE 2:** Participants characteristics in resilient and non‐resilient for Site 1—Wake Forest University and Site 2—University of Pittsburgh.

	Site 1			Site 2		
	Mean ± SD or *n* (%)		*p*	Mean ± SD or *n* (%)		*p*
	Resilient (*N* = 11)	Non‐resilient (*N* = 10)		Resilient (*n* = 12)	Non‐resilient (*n* = 12)	
Age, years	73.36 ± 2.66	72.70 ± 2.63	0.57	73.75 ± 2.70	74.50 ± 4.12	0.60
Sex, women	2 (18%)	8 (80%)	0.009	5 (42%)	6 (50%)	0.68
Race, Non‐Hispanic White	9 (82%)	5 (50%)	0.18	11 (92%)	3 (25%)	0.003
Gait speed, m/s	1.80 ± 0.12	0.73 ± 0.09	< 0.001	1.3 ± 0.16	0.84 ± 0.12	< 0.001
Comorbidities, number	1.64 ± 0.67	2.00 ± 0.82	0.28	1.73 ± 0.79	2.36 ± 1.29	0.18
Time spent walking (min/week)	188.64 ± 282.03	168.00 ± 285.38	0.87	160.91 ± 181.12	21.70 *±* 45.41	0.03
Digit symbol substitution test, points	40.27 ± 9.69	27.90 ± 12.47	0.02	53.08 ± 7.50	17.42 ± 8.53	< 0.001
Modified mini‐mental status exam, points	92.82 ± 5.58	87.80 ± 5.33	0.05	96.50 ± 2.43	77.33 ± 4.46	< 0.001
Muscle strength, Nm	106.56 ± 19.42	61.08 ± 40.23	0.008	86.96 ± 36.38	78.51 ± 34.17	0.59

### 3.2 Characterization of Total Circulating Serum EV and Small ncRNA Cargos

We first characterized the isolated serum EVs based on size, concentration, protein content, and RNA concentration. NTA demonstrated enrichment of serum EVs of similar size in both the mobility resilient and non‐resilient groups (Figure 1A). NTA showed no significant difference in serum EV size between the groups, with most particles measured between 50 and 200 nm, which indicates enrichment of small EVs (Figure 1B). Similarly, there was no significant difference in EVs concentration between groups (Figure 1C). The enrichment of small EVs from serum samples was confirmed by Western blotting using canonical EVs markers CD63 and CD81 (Figure 1D). Furthermore, TEM further confirmed the presence of intact EVs isolated from serum, with enrichment of small EVs (Figure 1E). These findings demonstrate successful isolation and characterization of serum EVs from these participants, with no significant difference in their biophysical properties.

Following serum EVs characterization, total RNA was isolated with no significant difference in RNA amounts between groups (Figure 1F). Most of the isolated RNA displayed a size between 50 and 200 nucleotides, which is a characteristic of small ncRNA. The sequencing data were analyzed for the abundance of six distinct small ncRNA classes: circRNA, snoRNA, snRNA, miRNA (miR), piRNA, and tRNA annotated with COMPSRA (Li et al. 2020). We observed no differences between the groups in the number of annotated small ncRNAs identified in serum EVs (Figure 1G). These findings support that we efficiently isolated total circulating serum EVs and identified the small ncRNA cargos with no significant difference in the types or number of EVs or number of annotated small ncRNA cargos between the resilient and non‐resilient samples.

### 3.3 Differential Enrichment of Small ncRNAs From Total Circulating Serum EV of Mobility Resilient Individuals

**FIGURE 2:**
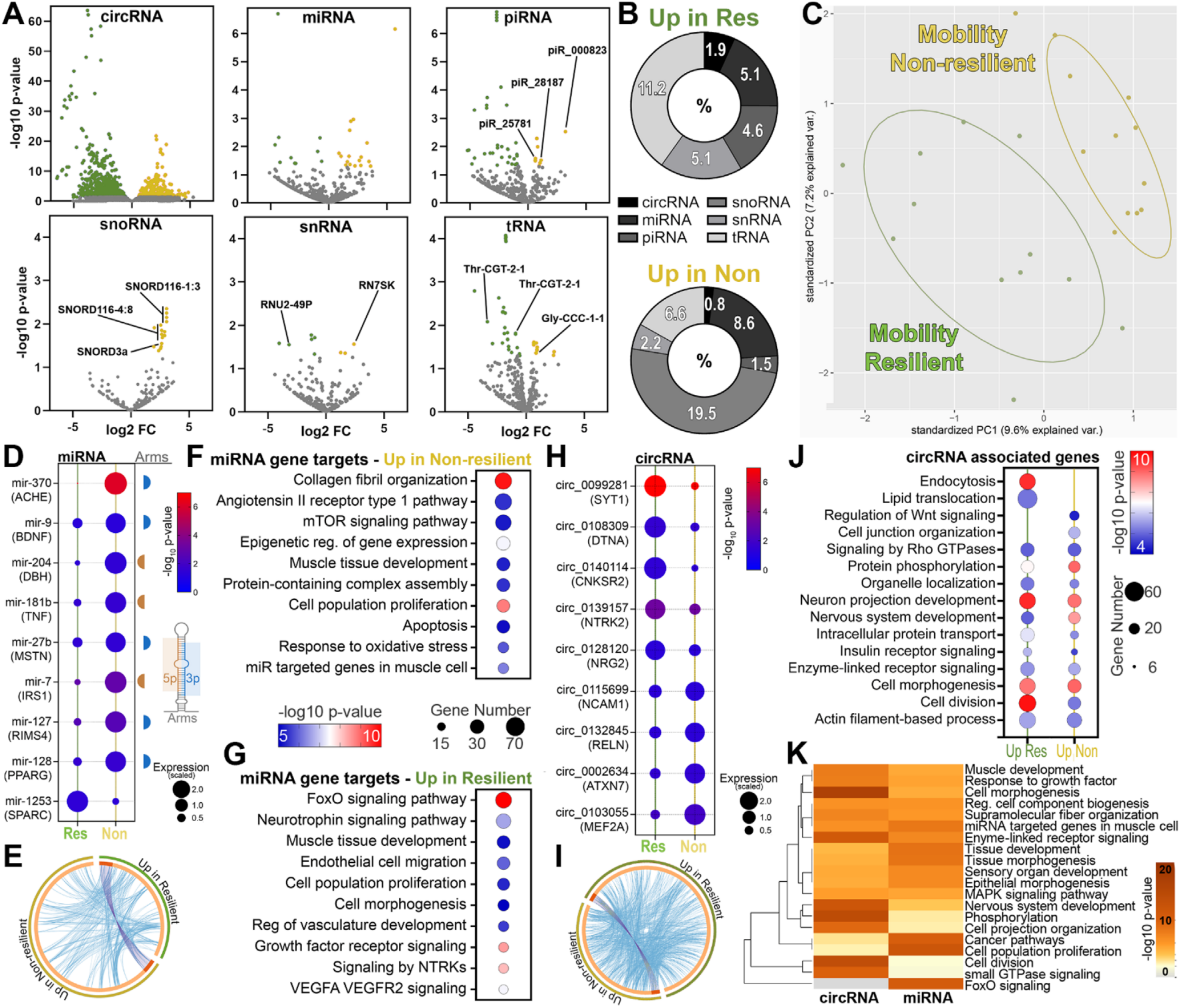
Unique small ncRNA profile of total circulating serum EVs associated with mobility status. (A) Volcano plots showing differential enrichment of small ncRNAs from total circulating serum EVs of resilient (Res) compared to non‐resilient (Non) individuals. (B) Donut plot showing distribution of significantly affected small ncRNA species compared to total annotated. (C) PCA plot showing unique small ncRNA profiles for the two groups. (D) Bubble plot showing abundance of selected significantly affected miRNAs, which are brain tissue enriched, except miRNA‐1253, which is muscle tissue enriched. Select target gene below the species and identified arm to the right of the plot. The color of the dot represents significance and size relative abundance. (E) Circos plot shows overlap of Targetscan defined target genes associated with miRNAs that were enriched in serum EVs of resilient or non‐resilient individuals. Dark orange color represents genes that are shared while light orange shows unique genes in the two groups. Purple lines link the shared genes, and blue lines link unique genes that belong to the same ontology term. (F, G) Bubble plots show the biological terms associated with the gene targets of enriched miRNAs in serum EVs of non‐resilient (F) and resilient (G) participants. The color of dot represents significance and size number of genes. (H) Bubble plot showing abundance of selected significantly altered circRNAs which show brain tissue enrichment. Selected associated gene below the species (I) Circos plot shows overlap of circBase defined circRNA associated genes that were enriched in the serum EVs of resilient or non‐resilient individuals. (J) Bubble plots showing biological terms of the associated gene of enriched circRNAs in serum EVs of resilient and non‐resilient participants. (K) Dendrogram of selected biological terms with the best *p*‐value that were common when assessing circRNA and miRNA associated input genes. The heatmap cells are colored by *p*‐value. *n* = 12.

Subsequent analysis on the differential abundance of the small ncRNA cargos was performed with negative binomial distribution (DESeq2) to determine statistical enrichment of these cargos in total serum EVs comparing resilient and non‐resilient individuals. Among the six classes of small ncRNAs, we found several species which were differentially abundant in the serum EVs between resilient and non‐resilient groups (Figure 2A). The circRNAs had the highest number of species which showed differential enrichment, with 720 increased in non‐resilient and 1588 increased in serum EVs from resilient participants (Figure 2A). In resilient individuals, there was a higher percentage of species significantly enriched belonging to tRNAs (11.2%), snRNAs (5.1%), and miRNAs (5.1%) while non‐resilient individuals, a high percentage was enriched in snoRNA (19.5%), miRNAs (8.6%) and tRNAs (6.6%) (Figure 2B). To reduce the dimensionality of the data and visualize differences in the types and abundance of annotated small ncRNA cargos, we performed a principal component analysis (PCA). PCA plot of the small ncRNA abundance levels for the resilient and non‐resilient segregated into two distinct clusters, indicating unique abundance profiles between groups (Figure 2C). Many of the significantly differential abundant miRNAs (miR‐370‐3p, miR‐204‐5p, miR‐181b‐5p, miR‐127‐3p) in serum EVs were previously shown to have enriched expression in brain tissue (Figure 2D), as identified in published small ncRNA tissue atlases (Alsop et al. 2022; Keller et al. 2022; Smal et al. 2024). Furthermore, these miRNAs have key target genes associated with healthy brain aging. Interestingly, miR‐1253 which is enriched in serum EVs of non‐resilient group showed skeletal muscle tissue specific expression in the published atlases. This suggests that serum EVs as a source for assessing changes in small ncRNA related pathways in both brain and peripheral tissue.

To better understand the biological significance in these enrichment patterns in the serum EVs, we identified target genes of the differentially enriched miRNAs with TargetScanHuman. Using the 27 significantly affected miRNAs (10 up in resilient and 17 up in non‐resilient), we identified 249 target genes associated with the resilient group and 471 associated with the non‐resilient group, with 24 target genes common targets of significantly enriched miRNAs from either group (Figure 2E, purple lines). There was a low concurrence of functional overlaps among the miRNA target genes associated with the two cohorts, suggesting changes in unique biological functions between groups (Figure 2E, blue lines). We submitted the miRNA target genes associated with the resilient and non‐resilient groups to Metascape aiming to uncover the potential functional roles of these miRNAs. In the non‐resilient group, we observed biological functions of the target genes associated with collagen fibril organization, mTOR signaling, response to oxidative stress, cell proliferation and apoptosis, muscle tissue development, and miRNA targeted genes in muscle cells (Figure 2F). In resilient individuals, target genes were associated with FoxO signaling, neurotrophin and growth factor signaling, muscle and vascular development, and endothelial cell migration (Figure 2G). We also found several circRNAs that had differential abundance between the two groups which previous publications demonstrate have enriched expression in the brain (Figure 2H) (Rybak‐Wolf et al. 2015; Wu et al. 2020). We identified 304 associated genes from the enriched circRNAs of the non‐resilient group and 551 from the resilient group. There were 19 associated genes that were common, and the genes associated with the two groups showed a higher level of functional overlap compared to miRNA target genes, suggesting a more shared biological function between the circRNA cargos of the two groups (Figure 2I). The resilient group had unique biological functions from the circRNA associated genes linked to endocytosis and lipid transport while the non‐resilient group had unique functions linked to cell junction and Rho GTPase signaling. The circRNA associated genes from both groups shared a lot of biological functions such as organelle organization, nervous system development, insulin and enzyme linked signaling, and cell morphogenesis and division (Figure 2I). Comparative analysis of the miRNA and circRNA associated genes highlights the potential role of these cargos in biological functions and pathways that may contribute to the phenotypes of the resilient and non‐resilient individuals (Figure 2K). The hierarchical term clustering demonstrates that serum EV cargos have shared roles in biological functions such as muscle and nervous system development, response to growth factors, cell projections, proliferation and morphogenesis, and cell component biogenesis. Overall, analysis of the small ncRNA cargo from serum EVs of resilient compared to non‐resilient group suggests packaging of these cargos is not random and may be involved in aspects of phenotypes, disease onset, and progression. These results also show a small ncRNA mobility‐related signature of serum EVs, which this analysis suggests capture changes in disease mechanisms related to brain and muscle functions.

### 3.4 MDE Display Unique Molecular Signatures of Mitochondrial Function According to Mobility Status

**FIGURE 3:**
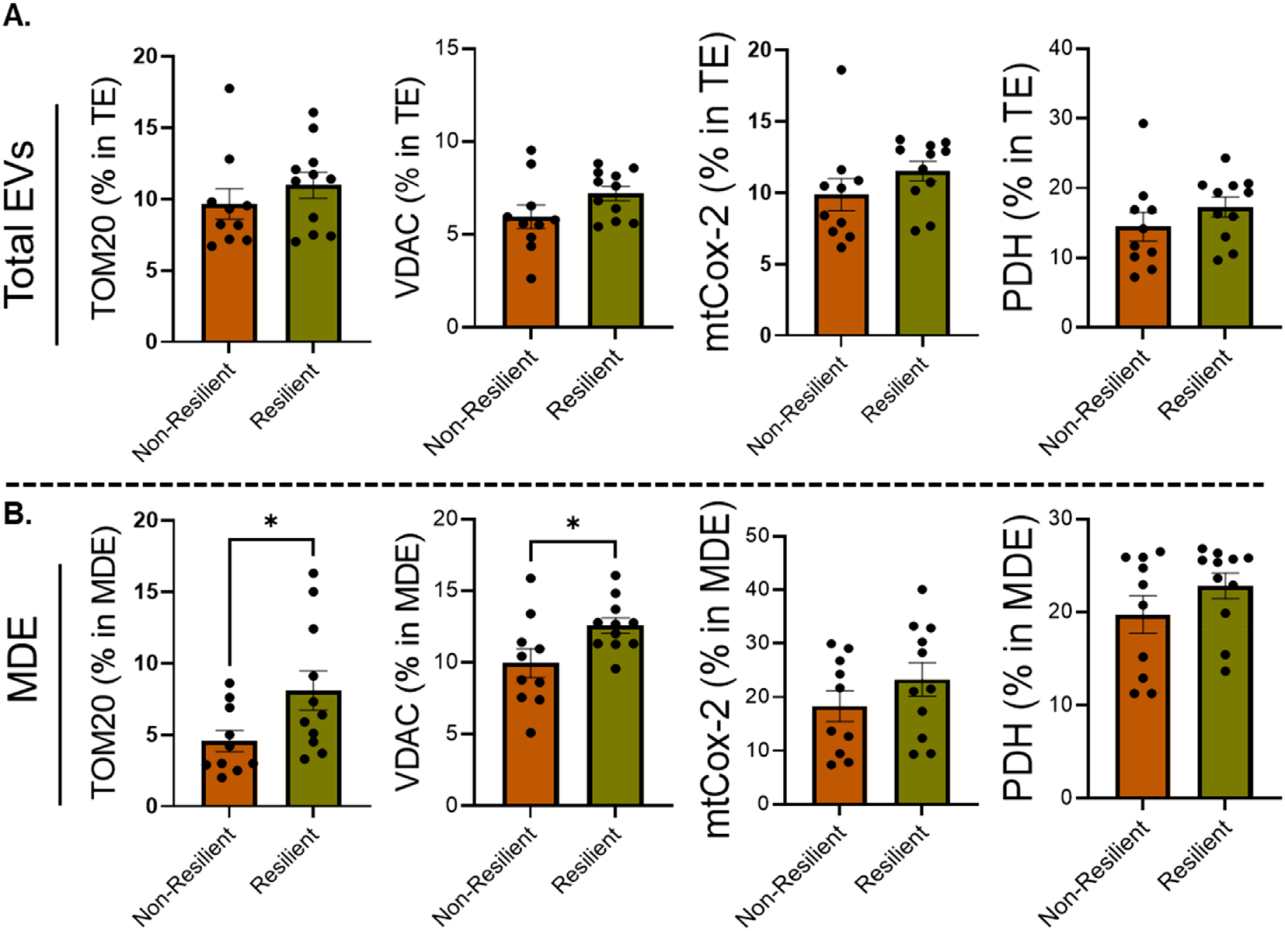
Assessment of mitochondrial markers in total serum EVs and muscle‐derived EVs (MDE) by nanoflow cytometry. (A) Serum samples, from mobility non‐resilient (*n* = 10) and resilient (*n* = 11) participants were processed to isolate total EVs (TE) (A) and MDE (B) and analyzed for the surface levels of TOM20, VDAC, mtCox‐2, and PDH. Statistical significance was determined by unpaired *t*‐test. *p < 0.05.

**FIGURE 4:**
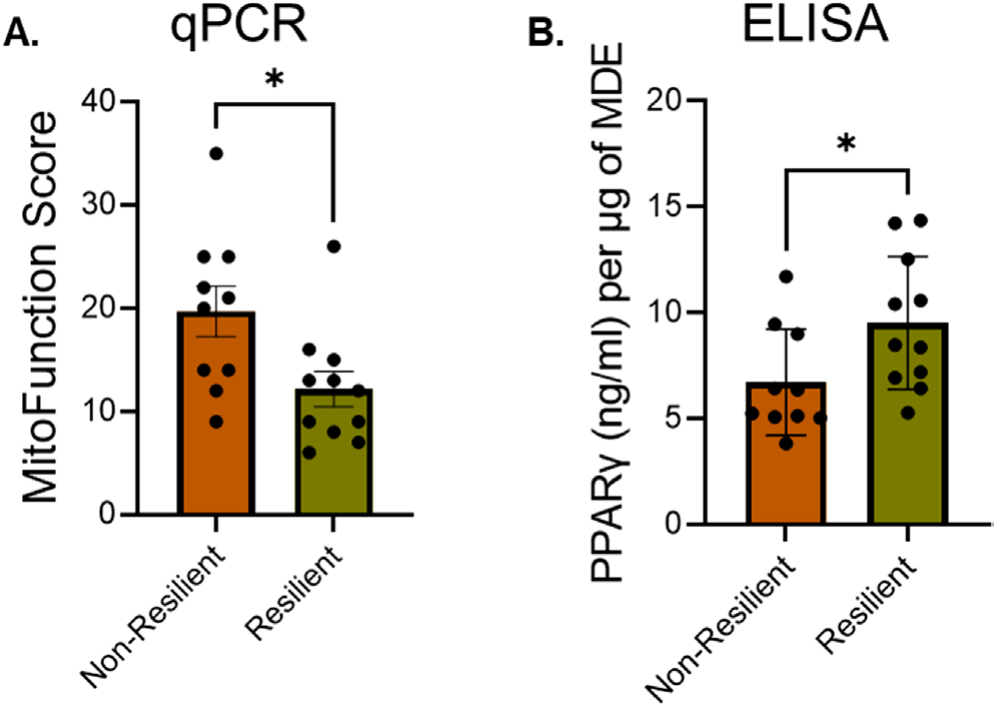
Assessment of MDE cargos for biomarkers of mitochondrial function. (A) MDE were analyzed for 13 miRNAs by RT‐PCR and based on their levels the MitoFunction Score calculated for non‐resilient and resilient groups as described in the methods. (B) Levels of PPAR‐γ were analyzed using ELISA in MDE from non‐resilient and resilient groups, after normalization with MDE proteins concentration. Statistical significance was determined by unpaired *t*‐test. *p < 0.05.

To better elucidate the ability of EVs cargo to reveal skeletal muscle ergogenic states, we isolated total circulating serum EVs from the serum Health ABC participants, followed by MDE isolation (Mishra et al. 2025). The nano‐flow cytometry analysis showed no significant difference in surface levels of mitochondrial markers TOM20, VDAC, mtCox‐2, and PHD (Figure 3A) in total circulating serum EVs between the groups. Importantly, the MDE isolated from resilient samples displayed higher surface levels of mitochondrial markers, including TOM20 and VDAC, compared to the non‐resilient samples (Figure 3B). However, we did not observe any significant difference in the surface levels of mtCox‐2 and PDH between groups in either total circulating serum EVs or MDE. Next, we characterized the MDE cargos to analyze the level of miRNAs and MitoFunction Score, as described in the methods. First, we analyzed the level of a panel of 13 miRNAs, regulating mitochondrial function to determine their association with mobility resilience, in MDE. Second, the MitoFunction Score was calculated, based upon the change in levels of these miRNAs (Figure S1), which displayed significantly higher score for non‐resilient as compared to resilient individuals (Figure 4A), suggesting a greater alteration in the level of these miRNAs in non‐resilient individuals. At last, we observed higher levels of PPAR‐γ in the MDE of resilient group (Figure 4B); a nuclear receptor, which when activated promotes mitochondrial biogenesis (Corona and Duchen 2016).

Using linear regression modeling, we associated levels of the assessed MDE cargos with clinical phenotypes. Age and gender adjusted associations between these MDE cargos and gait speed were statistically significant for TOM20, miR34a‐5p and miR27b‐3p (adjusted coefficients [*p* values]: 0.50 [*p* = 0.028], −0.67 [*p* = 0.002], −0.50 [*p* = 0.03]), but not PPAR‐γ. Additionally, TOM20 and miR34a‐5p were also associated with muscle strength (adjusted coefficients [*p* values]: 0.58 [*p* = 0.03], −0.73 [*p* = 0.003]). TOM20 was positively associated with 3MS score (adjusted coefficients [*p* values]: 0.49 [*p* = 0.033]) and miR34a was negatively associated with DSST cognitive score (adjusted coefficients [*p* values]: −0.57 [*p* = 0.014]).

## 4 Discussion

Our study is the first to provide a comprehensive characterization of total serum EVs and MDE in older adults with differing mobility status. By integrating small ncRNA profiling and mitochondrial marker analysis, we demonstrate that EVs carry distinct systemic and muscle‐specific signatures reflective of biological aging and functional capacity. The identification of differentially enriched miRNAs, circRNAs, and mitochondrial proteins in EVs from resilient versus non‐resilient individuals highlights their potential as non‐invasive biomarkers of mobility outcomes. We found that small ncRNA cargos were differentially abundant in older adults who were resilient compared to non‐resilient, with the highest differences in tRNAs, miRNAs, and snoRNAs. The associated genes of differentially abundant miRNAs and circRNAs indicated variations in biological processes related to muscle and nervous system development, metabolic signaling, and cellular responses to stress. Focused analysis of MDE exhibited enhanced mitochondrial markers in resilient individuals, suggesting a role in preserving muscle bioenergetics. Our study demonstrates the possibility of small ncRNA and protein EV cargos as biomarkers for mobility resilience in older adults which are associated with biological functions important in muscle and brain health. This work advances the field of precision geroscience by linking EV mediated molecular communication to functional aging phenotypes and establishes future biomarker‐driven strategies to assess mobility resilience and healthy aging. These results also set a foundation for future mechanistic studies examining how total serum small ncRNA cargos and MDE cargos can functionally impact mobility resilience in aging individuals.

Our initial characterization of total circulating serum EVs revealed no significant differences in size, concentration, or canonical EV markers between resilient versus non‐resilient groups. These findings suggest that the observed molecular differences are not due to EVs quantity or quality, but rather a selective cargo loading. Further, small ncRNA profiling identified a diverse repertoire of RNA species, consistent with recent reports highlighting their enrichment in circulating EVs and roles in aging and cellular stress responses (Abramowicz and Story 2020; Chen et al. 2024; Shin et al. 2024). Interestingly, in the resilient group, 8 of 17 significantly differential abundant miRNAs show higher levels in the brain compared to other tissues according to small ncRNA tissue atlases (Alsop et al. 2022; Keller et al. 2022; Smal et al. 2024). This contrasts with the non‐resilient group, which had no increased miRNAs that show brain‐enriched expression. Furthermore, miR‐1253, which is highly expressed in muscle tissue and has been shown to impair proliferation and induce apoptosis (Wang et al. 2020), was significantly increased in the total circulating serum EVs of non‐resilient individuals. This highlights the importance of miRNA cargos in the communication of brain and peripheral tissues and could provide a mechanism for mobility resilience with age.

Impaired metabolic flexibility of skeletal muscle likely contributes to the development of certain chronic diseases including obesity and type 2 diabetes, in addition to affecting mobility resilience with age (Goodpaster and Sparks 2017). In our data, miR‐183, a master regulator of metabolic homeostasis in skeletal muscle, was enriched with the highest fold change in serum EVs isolated from non‐resilient individuals. Increased miR‐183 level could decrease β‐oxidation and lipolysis in skeletal muscle and facilitate glucose utilization by impairing phosphorylation of PDHA1 via targeting FoxO1 (Wang et al. 2021). Furthermore, previous studies have shown that decreasing miR‐183 enhances fat catabolism in skeletal muscle and has a beneficial effect for muscle health after a high fat diet challenge (Wang et al. 2021). It is generally accepted that decreased β‐oxidation and higher utilization of glucose stores in skeletal muscle are associated with metabolic diseases (Koh et al. 2019; Sanchez‐Gonzalez et al. 2020), which, along with changes in lipid metabolism, could contribute to mobility decline in non‐resilient individuals.

Interestingly, we observed significant increases in miR‐3168 and miR‐126 in EVs of non‐resilient individuals, which may impact vascular health contributing to mobility phenotype. Overexpression of miR‐3168 and miR‐126 impairs angiogenesis by targeting *BMPR2* (Lago‐Docampo et al. 2024) and *HIF‐1α* (Alique et al. 2019), respectively. Crosstalk between skeletal muscle and vascular endothelial cells is essential for the coordinated delivery of oxygen and nutrients. This interaction, coupled with angiogenic signaling, enhances capillary density and perfusion, thereby supporting improved metabolic efficiency. This link between skeletal metabolic functions and miRNA EV cargos is further exemplified by significant increases in miR‐29a‐3p in total circulating serum EVs of resilient individuals. The level of miR‐29a‐3p in total circulating serum EVs is increased after resistance training and mediates changes in muscle energy metabolism (Pinto‐Hernandez et al. 2025). There is strong evidence that mitochondrial dysfunction leads to alterations in skeletal muscle metabolism, a hallmark of aging and a root cause of progressive loss of skeletal muscle function and CNS dysfunction, both of which contribute to mobility decline and ultimately physical disability (Tian et al. 2023).

In total circulating serum EVs, the abundance of tRNAs was also significantly impacted by mobility resilience in older individuals. It remains largely unknown how physiological and pathological conditions regulate tRNAs and their derivatives, which are important components of the protein synthesis machinery in muscle. Of particular interest, two tRNAs, Thr‐CGT‐2‐1 and Tyr‐GTA‐9‐1, were enriched in resilient individuals. These tRNAs have previously been associated with better muscle strength and physical performance in older adults (Shin et al. 2024), suggesting their potential role in promoting mobility resilience. Interpretation of these findings could indirectly be linked to anabolic effects and muscle protein synthesis given the critical role of leucine (Leu). Notably, Leucyl‐tRNA synthetase is an intracellular sensor that transfers Leu to cognate tRNAs to form tRNA‐Leu species and this activates mTOR signaling, serving to activate the protein synthesis and anabolic processes (Rehman et al. 2023). There are no studies that have directly investigated tRNA‐Leu species and muscle‐related metabolism, and therefore, the underlying mechanisms remain unclear. Our study is in agreement with a similar result that circulating tRNA‐Leu species are significantly affected in older individuals with low muscle strength and physical performance (Shin et al. 2024), whereas in our study, we demonstrate that the source of these circulating tRNAs are the serum EVs.

Mobility decline with age is the result of the interplay of progressive loss of skeletal muscle function, CNS dysfunction, and altered brain‐muscle communication. We detected several snoRNAs and piRNAs cargos that were significantly enriched in total circulating serum EVs from the non‐resilient group, that have been previously linked to neurodegeneration. Several members of the SNOD116 family were increased in non‐resilient individuals. SNORD116 is predominantly expressed in the brain, and we previously demonstrated increased levels of members of the SNORD116 family in the plasma EVs of AD patients (Fitz et al. 2021). SNORD116 functions by regulating the stability of mRNA targets such as Nhlh2, a transcription factor involved in energy homeostasis and metabolic function (Kocher et al. 2021). Similarly, we observed increased levels of piR_25781, piR_28187 and piR_28188 in the total circulating serum EVs of non‐resilient individuals. These piRNAs are increased in brain tissue of AD patients (Qiu et al. 2017; Roy et al. 2017). These results highlight that biomarkers associated with neurodegeneration are also increased in the total circulating serum EVs of non‐resilient individuals. This suggests the CNS may be the origin for these EVs and that alterations in brain EV cargos could contribute to changes in mobility through brain‐muscle cross talk. This also highlights a limitation of utilizing total circulating serum EVs as a liquid biopsy. Total circulating serum EVs contain EVs released by multiple organs, including the brain, where there are mechanisms allowing for the transport of EVs across brain barriers. Non‐resilient compared to resilient individuals in our study exhibited significantly worsened cognitive testing scores which could not only contribute to the unique small ncRNA signature observed from the total circulation serum EVs but also contribute to mobility decline with age. More research is needed to better define markers of brain released EVs found in peripheral biopsies that may contribute to brain‐muscle communication and associated with the pathology of mobility decline with age.

Skeletal muscle is one of the largest organs in the body and can release EVs into the circulating serum, which can play a significant role in communication of multiple systems. Recent work by Watanabe et al. showed that most skeletal muscle‐derived EVs in mice remain within the muscle interstitium and contribute minimally to circulating EVs (Watanabe et al. 2022). However, while most muscle EVs may act locally, a detectable subset enters the bloodstream for circulation. This is supported by Lin et al. (2026), who showed that exercise is able to significantly impact the release and cargos of skeletal muscle‐derived EVs, and that these circulating EVs are able to cross the brain barriers to modulate microglial functions, potentially serving as a protective mechanism in preclinical Alzheimer's disease mouse models. Using SGCA‐immunocapture, which we have extensively validated in prior work (Mishra et al. 2025), we enriched this circulating fraction and identified mitochondrial proteins and miRNAs associated with mobility phenotypes. These results suggest that even a small circulating population of muscle‐derived EVs can reflect muscle metabolic status and provide meaningful biomarker information. Further, we demonstrated that EVs derived from muscle contain not only differential abundant miRNAs that are important in mitochondrial function but also display changes in protein cargoes that are important in muscle metabolic functions. In MDE, resilient individuals showed significantly higher levels of surface mitochondrial markers, indicating enhanced mitochondrial content and potential bioenergetic capacity. This finding aligns with the concept that mitochondrial health is a key determinant of muscle function and resilience in aging (Amorim et al. 2022; Joseph et al. 2016; Picca et al. 2018). MDE could serve as a vehicle for mitochondrial proteins in intercellular communication, delivering metabolic signals that support tissue homeostasis and adaptation to stress. Importantly, the levels of both miR‐27b‐3p and miR‐34a‐5p in MDE showed a strong negative association to mobility phenotypes (gait speed and muscle strength). EV‐associated miR‐34a is a well‐established senescence‐related miRNA, and senescent skeletal muscle cells secrete higher levels of EV‐associated miR‐34a as part of the senescence‐associated secretory phenotype, a pattern observed in both human and animal studies (Alfonzo et al. 2022). This aligns with our finding that elevated MDE miR‐34a is associated with poorer mobility and suggests that miR‐34a‐positive MDE may reflect an increased senescence burden in aging muscle. Recognizing this connection also highlights potential therapeutic directions that reducing senescent cell load or targeting miR‐34a regulatory pathways, including enhancing MALAT1, a competing endogenous RNA that limits miR‐34a activity (Ruan et al. 2021), may offer avenues to mitigate mobility decline in older adults. Notably, prior work also shows that muscle‐derived, SGCA‐positive EVs exhibit increased miR‐34a with age in mice (Fulzele et al. 2019), supporting a conserved link between EV‐miR‐34a and age‐related musculoskeletal decline. Moreover, this cross‐species consistency supports the biological relevance of our findings and reinforces the use of mouse models for mechanistic studies related to mobility decline.

Further analysis of the MitoFunction Score demonstrated an increase in non‐resilience individuals. Since the MitoFunction score is not based on the directionality of change in levels of its contributing miRNAs, its increase in non‐resilience individuals may reflect a compensatory response to mitochondrial dysfunction, as mitochondrial‐related miRNAs are known to play an important regulatory role in conditions of oxidative stress and impaired bioenergetics (Luo et al. 2023). Additionally, higher levels of PPAR‐γ in MDE from resilient individuals suggest a state of relatively higher mitochondrial biogenesis. Interestingly, we also observed that two of the miRNAs which were enriched in total circulating serum EVs from non‐resilience individuals have target genes that are associated with *VDAC* (miR‐183‐3p) and *PPARG* (miR‐27b‐3p), suggesting that in individuals with declining mobility, not only could these protein cargos be diminished in EVs but expression of their associated genes could be repressed by these miRNA cargos in target tissues. Importantly, we observed associations in the levels of TOM20 in MDE with mobility phenotypes in this cohort. These associations between protein cargo and previously reported associations with miRNA cargo underscore the potential of MDE as biomarkers for mobility decline. Interestingly, we also observed associations between TOM20 and miR‐34a‐5p and cognitive assessments in this cohort, which supports the idea that EVs of muscle origin can modulate cognitive functions in older adults. To validate and expand upon these findings, further investigation of MDE cargo should be conducted in a larger, well‐characterized cohort. These findings also support the hypothesis that MDE carry molecular signatures indicative of skeletal muscle ergogenic states and that these signatures differ according to mobility resilience. The enhanced mitochondrial marker levels in resilient individuals may reflect preserved mitochondrial integrity and adaptive capacity, contributing to better physical performance.

The results of this study demonstrate that total circulating serum EVs from resilient older adults have a unique small ncRNA signature related to genes important in brain and muscle development, metabolic homeostasis, and cellular response to stress. Further MDE revealed differential levels of TOM20, VDAC, and PPAR‐γ as well as MitoFunction score between the two groups. In addition, TOM20, miR‐34a‐5p, and miR‐27b‐3p isolated from MDE showed a strong association to mobility phenotypes. Although the outcomes of much larger independent validation studies are difficult to predict, the results presented here point to a newly discovered association of EVs cargos with mobility resilience in aging individuals. This preliminary study supports a large cohort study examining the potential of total circulating serum EVs or EVs from brain and muscle origin as a novel source in search of biomarkers with high discriminatory power to differentiate changes in mobility associated with age. Moreover, the EV cargos offer a valuable source for understanding age‐associated mechanisms and monitoring biological processes impacted by interventions or therapies to improve mobility resilience.

Together, our data suggest that total circulating serum EVs and MDE provide complementary insights into the biological underpinnings of mobility resilience. The small ncRNAs from serum EVs reflect systemic and CNS‐related pathways, whereas MDE reveals muscle‐specific mitochondrial signatures. Integrating both the molecular profiles offers a powerful approach to identify biomarkers and therapeutic targets for promoting resilience in aging. Moreover, the identification of specific ncRNAs and mitochondrial markers associated with resilience opens avenues for targeted interventions. For example, enhancing the levels of protective tRNAs or circRNAs, or modulating miRNA networks involved in FoxO and neurotrophin signaling, may improve muscle and vascular health. Similarly, strategies to boost mitochondrial content in MDE, such as exercise, nutritional supplementation, or pharmacological activation of PPARγ, could enhance muscle bioenergetics and delay mobility decline. This study demonstrates that total circulating serum EVs and MDE carry molecular signatures reflective of mobility resilience in older adults. The differential enrichment of small ncRNAs and mitochondrial markers between resilient and non‐resilient groups highlights the potential of EVs as biomarkers and modulators of aging‐related functional outcomes. By elucidating the molecular cargo of EVs, we pave the way for novel diagnostic and therapeutic strategies to promote healthy aging and preserve mobility.

## Author Contributions

Conceptualization, N.F.F., C.R., F.A., G.D.; methodology, N.F.F., A.K., G.D.; investigation, N.F.F., A.K., Y.S., M.S., S.S.; formal analysis, N.F.F., A.K., C.R.; visualization, N.F.F., A.K., C.R.; writing – original draft, N.F.F., A.K., C.R., G.D.; writing – review and editing, N.F.F., A.K., Y.S., M.S., S.S., R.K., I.L., A.S., F.A., C.R., G.D.; supervision, N.F.F., C.R. G.D.; project administration, N.F.F., C.R. G.D.; resources, N.F.F., C.R. G.D.; funding acquisition, N.F.F., C.R. G.D.

## Funding

This study was supported by National Institutes of Health R01AG075069 (NFF): R01AG075992 (IL,RK): R01AG068629 (GD): 1R01AG084696 (GD): N01‐AG‐6‐2101 (CR): N01‐AG‐6‐2103 (CR): N01‐AG‐6‐2106 (CR): R01AG028050 (CR): R01NR012459 (CR): Pittsburgh Foundation: Pittsburgh Pepper Center Grant Developmental Project (P30 AG024827).

## Ethics Statement

All studies were approved by the University of Pittsburgh Institutional Review Board.

## Conflicts of Interest

G.D. is the founder of LiBiCo LLC, which has no influence or contribution to the work presented in this manuscript. Other authors have no conflicts of interest.

## Supporting information


**Figure S1:** acel70470‐sup‐0001‐FigureS1.docx.

## Data Availability

Raw small noncoding RNA sequencing data were submitted to GEO (https://www.ncbi.nlm.nih.gov/geo/) and will be publicly available as of the date of publication. This paper does not report the original code. Any additional information required to reanalyze the data reported in this paper is available from the corresponding author upon request.
