# Supplementary material for: Serum‐Derived Extracellular Vesicles as Biological Indicator of Mobility Resilience in Older Adults
Source: Aging Cell. 2026 Apr 6;25(4):e70470. doi: 10.1111/acel.70470 (PMC13052232; doi:10.1111/acel.70470)
Supplement: Supplementary file 1 — Figure S1: acel70470‐sup‐0001‐FigureS1.docx. [file ACEL-25-e70470-s001.docx]

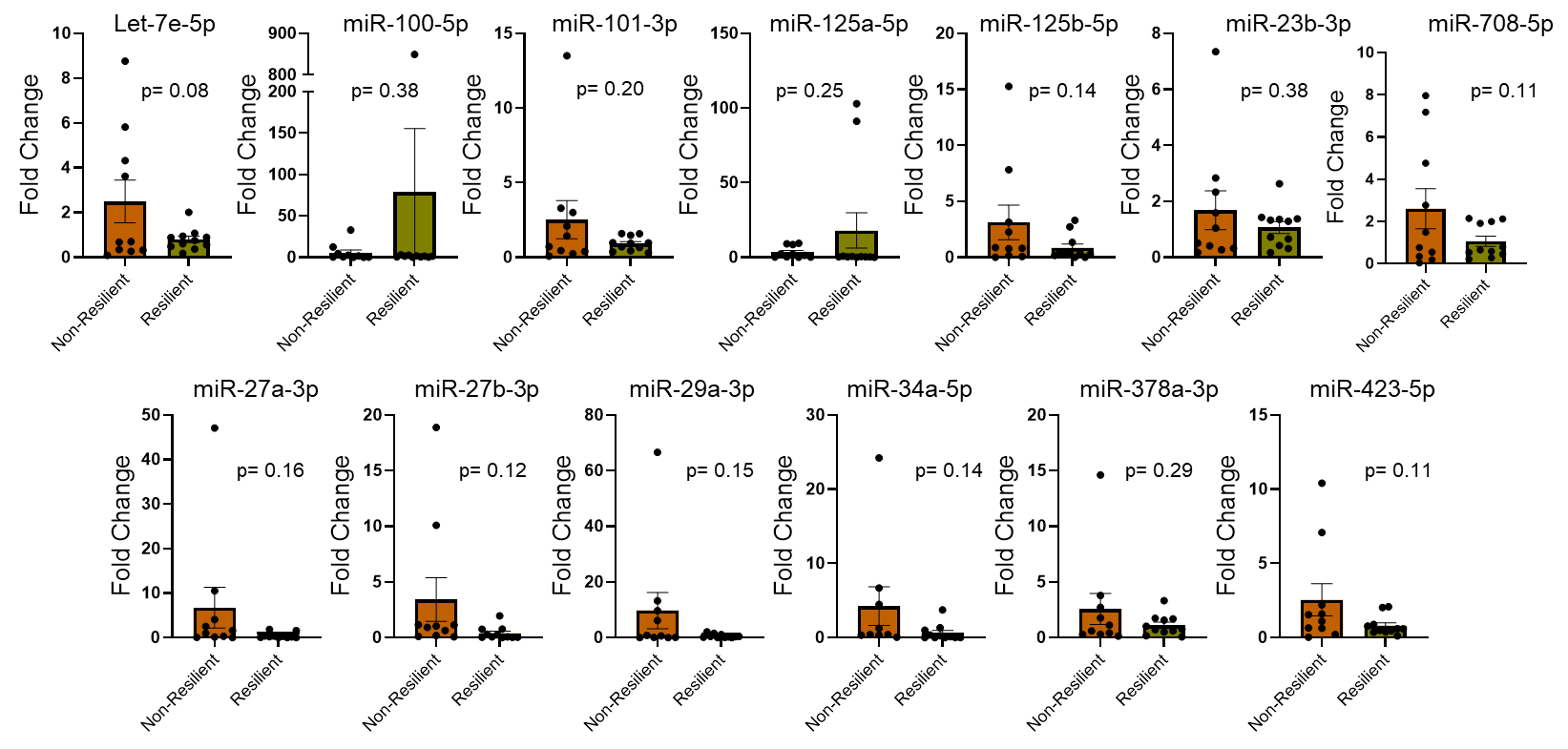
Supporting Information

**Supplemental Figure 1.** The expression of 13 miRNAs was analyzed in MDE isolated from mobility resilient and non-resilient groups by RT-PCR. cel-miR-39 was used to normalize the expression of miRNAs as described in methods, and fold change was calculated using non-resilience group as reference. Statistical significance was determined by unpaired t-test and p values are mentioned for each miRNA.
